# Motives and Barriers to Physical Activity Participation Among University Students

**DOI:** 10.3390/ijerph22111646

**Published:** 2025-10-29

**Authors:** Sami Elmahgoub, Hassan Mohamed, Adel El Taguri, Tamás Beregi, Aseel Aburub, Pongrác Ács

**Affiliations:** 1 Department of Physiotherapy, Faculty of Allied Medical Sciences, Applied Science Private University, Amman 11931, Jordan; a_abualrob@asu.edu.jo; 2 Department of Sport Training, Faculty of Physical Education and Sport Sciences, University of Tripoli, Tripoli 13932, Libya; has.mohamed@uot.edu.ly; 3 Department of Community Medicine, Faculty of Medicine, University of Tripoli, Tripoli 13932, Libya; tajoury@doctor.com; 4 Doctoral School of Health Sciences, Faculty of Health Sciences, University of Pécs, H-7621 Pécs, Hungary; tamas.beregi@etk.pte.hu; 5 Institute of Physiotherapy and Sports Science, Faculty of Health Sciences, University of Pécs, H-7621 Pécs, Hungary; pongrac.acs@etk.pte.hu

**Keywords:** physical activity, motives, barriers, university students, participation

## Abstract

**Background:** Physical activity (PA) is essential for the overall physical, mental, and psychological health of university students. However, participation rates remain low, particularly in developing regions such as Libya. **Materials and Methods:** This cross-sectional study involved 768 university students who completed self-administered questionnaires assessing PA engagement, motivations, and barriers to participation. **Results:** The findings revealed that 60.5% of participants were physically inactive, highlighting a significant public health concern. Among inactive participants, the most prevalent external barriers were time constraints (3.45 ± 1.07), limited resources (3.22 ± 0.97), and lack of support (2.96 ± 1.01). values are presented as mean ± standard deviation (SD). The primary motivating factors for PA were health improvement (3.95 ± 0.96) and revitalization (3.93 ± 1.01). Notably, gender differences emerged: male students reported higher motivation for competition and enjoyment, while female students were primarily motivated by weight management. Furthermore, female students reported significantly higher scores for both internal and external barriers than their male counterparts, indicating greater challenges faced in engaging with PA. The study also found a pronounced decrease in PA levels during the college years, suggesting that the transition to university life contributes to reduced activity. **Conclusions:** This study highlights the critical need for focused strategies to increase student participation in PA and address the identified barriers. Understanding both motivational aspects and barriers is essential for promoting healthier lifestyles among university students in Libya, which could ultimately lead to better health outcomes and fostering a more physically active student community.

## 1. Introduction

Engaging in physical activity (PA) is essential for the overall physical, mental, and psychological well-being of university students [1,2]. Regular PA significantly enhances physiological efficiency, reduces health risks, improves daily functioning, and promotes well-being during a key developmental period [3,4]. It has also been shown to improve mood, enhance sleep quality, and reduce anxiety and stress levels [3,4]. To achieve these benefits, the World Health Organization (WHO) recommends that adults aged 18–64 accumulate at least 150 min of moderate-to-vigorous intensity aerobic activity per week to improve overall health and reduce the risk of chronic diseases and depressive disorders [5].

Despite these well-established benefits and clear guidelines, a significant proportion of the global population, including university students, fails to meet these recommendations. This state of not engaging in sufficient PA is defined as physical inactivity (PIA) [6]. PIA is itself considered a leading public health issue of the 21st century [5] and is a particular concern in developing countries [7]. According to the WHO, approximately 3.3 million deaths annually are attributable to PIA globally, ranking it as the fourth leading risk factor for mortality worldwide [8]. This underscores the critical disconnect between knowledge of PA’s benefits and actual behavior, a gap that is especially pronounced among student populations [9,10,11].

Motivation is a fundamental psychological concept that influences behavior across various aspects of life domains, including daily needs, learning, work, and the pursuit of personal objectives [12]. A key distinction in psychology is between intrinsic motivation (IM) and extrinsic motivation (EM) [13,14]. IM refers to engaging in an activity for the inherent satisfaction and enjoyment of the activity itself, such as a student who exercises because they find the sport fun or personally challenging. In contrast, EM refers to engaging in an activity to attain a separable outcome, such as exercising to improve physical appearance, receive social recognition, or comply with a doctor’s advice. Motives for PA have been theorized by differentiating between the IM achieved through practice and the EM obtained from the outcomes of that practice [15,16]. The diverse motivational factors driving PA participation are often countered by significant barriers. Common obstacles prevent individuals from engaging in PA; the most common reasons are lack of sufficient time, discomfort with PA, lack of motivation, and fear of injury [17]. Understanding the interplay between motivation and barriers is crucial for promoting PA among university students. Motivation serves as the internal drive that encourages engagement, while barriers represent external obstacles that hinder participation. This relationship can be viewed as a dynamic push-and-pull interaction [13]. Health promotion motives, including the desire to maintain and enhance physical health, often compete with external obstacles such as time constraints, lack of access to appropriate exercise facilities, and insufficient social support. Emotional motives, particularly the enjoyment derived from PA, can also be undermined by environmental barriers that discourage engagement. For example, female students may face unique challenges related to societal expectations and limited access to safe exercise spaces, which can reduce their participation rates [11]. However, the presence of barriers such as limited information about the benefits of exercise or fear of judgment can significantly diminish both types of motivation. Research indicates that while students may be motivated by health and fitness goals, they often encounter psychological barriers that hinder their ability to engage in regular PA [18].

Barriers to PA are multifaceted and can be conceptualized using a framework based on the Social-Ecological Model [19], which considers influences at multiple levels. In this study, barriers are categorized into three broad, interacting levels: individual (e.g., psychological, cognitive), behavioral (e.g., activity traits, personal habits), and environmental (e.g., social, economic, physical environment). These are further detailed into six specific classifications: (1) Social, economic, and demographic factors; (2) Psychological, emotional, and cognitive factors; (3) Social and cultural factors; (4) Environmental factors; (5) Characteristics of physical activity; and (6) Behavioral traits [17]. This three-level classification elucidates the multifaceted nature of barriers. Individual factors include psychological, emotional, and cognitive influences that affect a person’s motivation and ability to engage in PA. Behavioral factors encompass characteristics of PA and individual behavior traits that impact participation. Environmental factors consist of social, economic, demographic, and cultural influences that shape the external context for PA. Multiple factors influence PA behavior, so investigating these aspects is important, especially in individuals in the late teenage period and young adults [20]. For example, the lack of suitable facilities, especially for female students, and the absence of supportive social networks are critical factors that deter students from participating in PA. Furthermore, they suggest that inactive college students report encountering more barriers than their physically active peers do, highlighting the importance of understanding these differences in promoting engagement.

Moreover, research has consistently demonstrated differences between genders in PA levels, motivations, and barriers among university students. Findings on activity levels have been mixed; some studies indicate that female students are more active than their male counterparts [21], whereas others report the opposite [22]. However, gender contributes significantly to both motivation for and barriers to PA participation. Males are more motivated by IM [23] and often pursue competitive aspects of sports and physical fitness, whereas females report significantly greater barriers [24] and may emphasize EM, including social aspects and personal well-being [23]. This distinction is crucial for adapting interventions that tackle unique needs and obstacles faced by different demographics [18].

Understanding the complex interplay between motives for and barriers to PA participation is important for implementing effective interventions. To design interventions that truly work, it is essential to understand the very human tug-of-war between the reasons students *want* to be active and the very real obstacles that are in their way. Research has emphasized the importance of intrinsic motivation, particularly when linked to enjoyment and personal fulfillment, in fostering long-term adherence to PA. However, this motivation can be easily undermined by external factors, necessitating a multifaceted approach to address both motivational and barrier-related challenges. Research indicates that motivating factors, such as the desire for improved health and enjoyment, are essential for encouraging PA [25]. However, these motivations often coexist with barriers like time constraints and lack of resources, which can impede action [11,20]. For example, a motivated student may face challenges that prevent them from participating, highlighting the need for interventions that address both aspects. A student might be deeply motivated by the desire to feel less stressed or to improve their health yet still find themselves inactive because their schedule is overwhelming or they lack a safe, accessible place to exercise [10,20,25]. This is the complex interplay between motivation and barriers: strong intentions are often quietly undermined by daily challenges. Furthermore, this balance is different for everyone. For example, a student’s gender can profoundly shape their experience, influencing everything from what motivates them to the specific social and environmental hurdles they must overcome [22,25]. Therefore, effective strategies must do more than just promote the benefits of physical activity; they must also thoughtfully address the specific barriers that prevent students from acting on their motivations.

Although considerable research has explored the motives for and barriers to PA participation among university students globally, notably, no studies have specifically examined this issue within the context of Libyan universities. Consequently, there is limited understanding of the unique factors driving PA engagement and the obstacles faced by students in Libya. Examining this relationship is essential for encouraging healthier lifestyles. Therefore, this study aims to examine the motives for and barriers to PA participation among Libyan university students and to explore how these factors vary by gender and academic discipline.

## 2. Materials and Methods

### 2.1. Participants and Ethical Considerations

This cross-sectional study was conducted at the University of Tripoli, Libya. A convenience sampling strategy was employed, which could limit wider applicability and introduce selection bias risk, yet the sample size used in this study was adequate for statistical evaluation. This allows for the exploration of motives and barriers associated with PA participation. Participants were recruited in person by the research team through the on-site distribution of questionnaires in common areas (e.g., libraries, student cafeterias) and outside lecture halls across various faculties. A total of 900 university students from this university were invited to participate in this study. The inclusion criteria for the study were: (1) being a currently enrolled undergraduate student at the University of Tripoli, and (2) being 18 years of age or older. The sole exclusion criterion was an inability or unwillingness to provide verbal informed consent to participate. Of these, 768 university students completed and returned the questionnaire. On the basis of their replies to a screening question about their engagement in PA (Do you practice physical activity?), participants were divided into two groups, and 303 participants who answered “YES” were identified as physically active. The remaining 465 participants who answered “NO” were identified as physically inactive. The participants’ ages ranged from 18 to 28 years; the sample comprised 496 females (64.6%) and 272 males (35.4%). Furthermore, participant recruitment took place in different faculties at the University of Tripoli, and the recruited sample was divided into three groups: health sciences students (55.9%), sciences and engineering students (21.6%), and humanities students (22.5%). This varied participant group aimed to accurately reflect the motives and barriers that university students face when engaging with PA. Ethical approval for this study was obtained from the Bioethics Committee at the Biotechnology Research Center (BEC-BTRC 2-2021), confirming that the research complied with ethical guidelines and standards for studies involving human participants. Informed consent was obtained from all participants, ensuring confidentiality and respect for their rights throughout the research process.

### 2.2. Data Collection

The study team handed out self-administered surveys to university students. Participants who answered ‘YES’ to the screening question about practicing PA were directed to complete the questionnaire on motives for PA participation. Conversely, participants who answered “NO” to the screening question about practicing PA were directed to complete the questionnaire related to the barriers to PA participation.

Data collection was conducted through the distribution of standardized questionnaires. Participants filled out the questionnaires independently, without direct interaction with investigators. This self-administered approach was specifically chosen to minimize social desirability bias and interviewer influence, as it allows participants to respond anonymously without the presence of an interviewer, thereby reducing the potential for them to give answers they perceive as socially acceptable rather than reflecting their true perceptions and behaviors. Participants were requested to fill out the questionnaire directly. Every submitted questionnaire was checked to make sure that all questions were answered.

### 2.3. Survey

All participants were asked to answer screening questions that included age, sex, faculty of study, and whether they were practicing PA.

Participants who answered ‘YES’ were asked to complete the Arabic version of the Exercise Motivations Inventory-2 (EMI-2) [26]. The EMI-2 is a widely used instrument with demonstrated reliability and validity for assessing a comprehensive range of motivations for exercise [26]. To the authors’ knowledge, it has been translated into Spanish, German, Hungarian, and Turkish. The Arabic translation was performed by Hashim Abdulla Al-Mousawi, from Arabian Gulf University, Bahrain, according to established international guidelines for cross-cultural adaptation [27]. The EMI-2 consists of 51 items divided into 14 subscales: Affiliation, Appearance, Challenge, Competition, Enjoyment, Health Pressures, Ill-Health Avoidance, Nimbleness, Positive Health, Revitalization, Social Recognition, Strength and Endurance, Stress Management, and Weight Management. Respondents answer each item on a 5-point Likert scale, ranging from 1 (‘not at all true for me’) to 5 (‘very true for me’). Each subscale includes 3 to 4 questions, and subscale scores are calculated by determining the mean of the relevant items as specified in the scoring key [26]. A higher mean subscale score indicates a stronger endorsement of that specific motive. Additionally, we incorporated further sociodemographic questions into the EMI-2, including sex, age, field of study, and year of study. We also added inquiries regarding the frequency of activity per week, the duration of PA sessions, whether individuals regularly engage in PA, and the onset of their PA practice. On average, it took participants about 10 to 15 min to complete the questionnaire.

In contrast, participants who answered ‘NO’ were asked to complete a 12-item barriers questionnaire developed by Arzu, Tuzun, and Eker (2006) [28]. This questionnaire has been used in prior research to identify obstacles to maintaining regular PA, and its utility has been supported in previous studies [28]. The questionnaire is divided into two main groups: the first six questions focus on internal barriers (energy-related obstacles, motivational factors, and self-confidence issues), and the second six questions examine external barriers (resource availability, social support, and time constraints). Responses were rated on a 5-point Likert scale from 1 (‘does not apply’) to 5 (‘strongly applies’). A higher mean score for a barrier item indicates that the barrier is perceived as a more significant obstacle to PA participation. On average, it took participants about 10 to 15 min to complete the questionnaire. The structured format and clear categorization of questions facilitate administration and analysis, supporting comparative studies and the development of effective strategies to promote PA.

### 2.4. Statistical Analysis

All data analyses were conducted using SPSS Statistics (Version 23.0; IBM Corp., Armonk, NY, USA). The score for motivations was calculated for the fourteen subscales. Scores on these subscales can range from 1 to 5, with higher mean scores indicating stronger motivation. These scores are presented as the means and standard deviations (mean ± SD). The basic descriptive parameters were calculated (mean, standard deviation, and frequency of answers). Independent samples *t*-tests were used to examine differences between the two sexes (male vs. female) for each item in the questionnaire. For comparisons across three or more groups (e.g., fields of study: health sciences, sciences and engineering, humanities), a univariate ANOVA with Bonferroni post hoc analysis was employed. Demographics (sex and field of study) served as the independent variables, and the 14 subscales were the dependent variables.

The barrier score was calculated for eleven subscales. Scores on these subscales can range from 1 to 5, with higher mean scores indicating that the barrier was perceived as more severe. These scores were subsequently summarized via descriptive statistics, specifically reporting the mean and standard deviation for each barrier. In addition to the average scores, basic descriptive parameters (including the mean ± SD), and frequency distribution of the responses for each questionnaire item were calculated. This provided a comprehensive overview of how participants rated various barriers to PA. Independent samples *t*-tests were used to examine differences between the two sexes (male vs. female) for each item in the questionnaire. A paired samples *t*-test was used to compare the overall internal and external barrier scores within the group of inactive participants. For comparisons across three or more groups (e.g., fields of study: health sciences, sciences and engineering, humanities), a univariate ANOVA with Bonferroni post hoc analysis was employed. Demographics (sex and field of study) served as the independent variables, and the 14 subscales were the dependent variables. Univariate ANOVA was used to assess whether there were statistically significant differences in barrier scores among the various demographic groups.

An alpha level of *p* < 0.05 was established to determine statistical significance, and the results were reported with 95% confidence intervals to provide an estimate of the precision of the mean scores. Overall, this rigorous statistical approach enabled the identification of significant trends and differences in motivation for PA participation and in the perceived barriers to PA among participants, thereby contributing valuable insights into the factors that influence PA engagement across different demographic segments.

## 3. Results

### 3.1. Demographic Characteristics of the Participants

Of the 768 questionnaires distributed, 465 participants (60.5%) were classified as physically inactive based on their negative response to the PA screening question, and 303 (39.5%) were classified as active. The majority of the participants were aged 20–25 years (77.9%) and were predominantly female (64.6%). Most students were classified as normal weight (69%), whereas approximately 24.4% were either overweight or obese. All participants’ demographic and occupational characteristics are summarized in Table 1.

### 3.2. The Motives for and Barriers to Physical Activity Participation of the Participants

Multivariate analysis (MANOVA) revealed significant overall effects of both sex (Pillai’s Trace = 0.36, F(14, 288) = 11.75, *p* < 0.001) and field of study (Pillai’s Trace = 0.18, F(28, 578) = 2.89, *p* < 0.001) on motivational profiles. In line with this, the most common motives for all participants were positive health and revitalization (3.95 ± 0.96; 3.93 ± 1.01, respectively), while health pressures and social recognition were the lowest (2.20 ± 1.18; 2.24 ± 1.09).

Follow-up univariate tests showed distinct motivational patterns. Male students reported significantly higher motivation than females in domains related to competence and social competition, including enjoyment, challenge, competition, social recognition, affiliation, and strength and endurance. In contrast, female students were significantly more motivated by weight management. Regarding the field of study, students in humanities scored higher on motives for Social Recognition and Affiliation, whereas students in sciences and engineering were more motivated by challenge compared to other faculties. All details are shown in Table 2.

### 3.3. The Barriers to Physical Activity Participation of the Participants

A MANOVA indicated that external barriers were significantly more prominent than internal barriers (3.21 ± 0.69 vs. 2.54 ± 0.62; *p* < 0.01), with ‘Lack of Time’ being the most common obstacle (3.45 ± 1.07). A significant multivariate effect of sex was found (Pillai’s Trace = 0.02, F(3, 460) = 2.93, *p* = 0.03), with females reporting significantly higher scores for a lack of resources (external) and a lack of energy (internal). In contrast, no significant multivariate effects were found for the field of study on perceived barriers. Consequently, female participants consistently reported significantly higher scores for both internal and external barriers than their male counterparts. A detailed description is provided in Table 3.

## 4. Discussion

This study highlights that the motives for PA participation differ based on gender and field of study. Male students are driven mainly by enjoyment and competition, whereas female students tend to focus more on weight management. Additionally, the findings reveal that barriers to PA participation also vary; female students encounter greater internal and external challenges, including time constraints, whereas male students generally face fewer obstacles. These results illustrate the complex interplay between motivation and barriers in PA participation, revealing that while students may have strong motivations, significant barriers often impede their engagement. This underscores the need for holistic health promotion strategies that enhance motivation while addressing the barriers students face to ensure more effective interventions.

Research on PA behaviors and barriers in Libya is limited, but growing. Current studies indicate that PA levels in Libya are typically low, with 70% to 75% of the population failing to meet the recommended PA guidelines [29]. The findings of this study align with the previously mentioned figure, indicating that approximately 60% of the participating university students were physically inactive. It should be noted that the “active” participants were not assessed for whether they met specific PA guidelines; thus, the true prevalence of inactivity may be even higher. This high percentage of physically inactive students is slightly higher than the figure for Jordanian students, where approximately 52% were reported as inactive [28], which corresponds to the documented levels of physical inactivity observed in countries of the Arab Gulf region [9]. In contrast, other Arab countries, such as Egypt and Lebanon, have shown significantly reduced levels of PIA among university students [5]. In addition, a study in Jordan reported a slightly lower percentage of inactive students (approximately 52%) [30]. Moreover, a systematic review reported that although differences in culture and education systems between different countries exist, university students have satisfactory PA levels [31]. This discrepancy highlights the varying cultural, social, and environmental factors influencing PA levels across the region. Common barriers to PA participation among Libyan adults include lack of time, limited facilities, and safety concerns [32]. Furthermore, cultural values are considered important barriers to PA participation for Libyan women, as they are linked to social expectations and a lack of opportunities for PA [33]. Additionally, to date, only one study has investigated the motives for PA participation among Libyan adults [34]. Most existing research on PA participation has focused on university or college students; however, neither the motives for nor the barriers to PA participation among university students in Libya have been investigated. This study, therefore, explored the motives for and barriers to PA participation and their differences based on sex and field of study among Libyan university students.

Overall, the participants in this study had both internal and external motivational factors contributing to their participation in PA. This finding aligns with results in the literature, where intrinsic and extrinsic motives have been linked to engagement in sports and exercise among college students [33,34,35]. The most important motivational factors for participating in PA were positive health, revitalization, strength and endurance, ill health avoidance, stress management, and weight management. These findings are in partial agreement with previous studies on university students [25,33,35,36] and Libyan adults [37] that used the same tool to measure motives for PA participation (EMI-2). Similarly, Malourinizi et al. used the Physical Activity and Leisure Motivation Scale (PALMS) to measure the motives for PA participation. They reported that maintaining physical health and relieving stress were among the top motivations reported by college students [23]. These results emphasize the role of both internal and external motives in engagement in PA among university students. The implementation of effective interventions that address specific motives could foster sustained PA participation, ultimately enhancing the overall well-being of university students.

In contrast, the key findings of this research showed that external barriers had a greater influence than internal barriers did on students’ participation in PA. These findings align with previous research on university students that emphasized the impact of a lack of time and environmental factors, such as access to facilities, social support, and community resources, on students’ engagement in PA [5,11,28,30,34,38]. However, research by DeSerio reported the opposite findings [18], where internal barriers (lack of energy and lack of willpower) significantly influenced PA participation among university students. Furthermore, according to a systematic review, the main barriers identified worldwide were a lack of time and motivation [20]. Addressing barriers to PA participation among university students, particularly the lack of time and resources, emphasizes the pressing demand for targeted efforts to tackle and overcome these barriers. To address this issue, university leaders should develop different strategies to enhance resources and facility access, and offer flexible PA programs and time schedules. This could promote an active lifestyle and improve health outcomes among university students.

Significant differences were found between males and females in their reasons for participating in PA. Our results revealed that five out of the 14 motives for PA participation, primarily internal motives (enjoyment, challenge, affiliation, competition, and social recognition), were significantly more common among male students than among female students. In contrast, female students reported a significantly greater motivation for weight management than male students. This aligns with previous literature [22,25,33,35,38,39,40]. For instance, Kilpatrick et al.’s study indicated that motives for PA participation varied by sex and PA type [33]. They reported gender effects for challenge, enjoyment, affiliation, and competition in males and weight management in females, similar to our results [33,35,36]. These findings highlight the importance of understanding the various influences that drive male and female students to participate in PA. Males often seek enjoyment, challenge, and competition, whereas females tend to focus on weight management. These unique motives can be used to design more personalized programs that resonate with each group to encourage PA and improve overall health.

On the other hand, female university students in this study reported significantly greater scores for both internal and external obstacles than their male counterparts did. These findings are consistent with those of existing studies [5,11,24,41]. Consistent with the overall trend, our study revealed that external barriers were rated significantly higher than internal barriers among the students, with ‘Lack of Time’ being the most prominent challenge. However, a nuanced finding was that the scores for lack of time were quite similar for both sexes. This aligns with the findings of some studies [41], but contradicts others, which report that females experience a significantly greater lack of time [18,24]. Furthermore, some research has shown that male students face a significantly greater lack of time barrier than females do [11,28], while another study found higher internal barriers among males [28]. These apparently conflicting findings reveal that female university students face greater internal and external barriers to PA than their male peers do. While both sexes report similar issues with a lack of time, there are mixed findings in the existing literature that suggest this barrier may not have a uniform gender association. This further underscores that gender-specific challenges should be considered when creating programs to encourage PA.

This study also identified differences in motivations for and barriers to PA participation among students from various fields of study. Specifically, students in humanities faculties reported significantly greater motives for competition, affiliation, and social recognition than students in other disciplines, while students in sciences and engineering reported a significantly greater motivation for challenge compared to other fields. Previous research on this topic has produced mixed findings. For example, Cerar et al. reported contrasting findings with significantly greater motivation related to weight management and appearance in social science students than in natural sciences students [40]. Yet, the same study also agreed with our results, indicating that the challenge motive was significantly greater among natural sciences students than among those of the social sciences discipline [40]. Regarding barriers, the pattern of external barriers being more prominent than internal ones was consistent across fields of study. These findings suggest that unique motivations for PA participation among different fields of study should be utilized to tailor PA program development in various faculties and universities. Furthermore, understanding the specific barriers faced by different fields of study can also help universities create targeted solutions. By focusing on what drives students, such as competition for humanities students or challenge for science and engineering students, universities can encourage more participation in PAs. The mixed nature of existing evidence highlights the need for further studies to better understand the varying motivations and barriers across academic disciplines.

### 4.1. Implications

This study highlights a pressing issue: the low levels of PA among university students, especially in Libya, where approximately 60% are inactive. This emphasizes the need to understand both the motives for students to be active and the barriers they face. Notably, male students often find motivation in enjoyment and competition, whereas female students are more concerned with weight management and encounter greater obstacles. To address these challenges, the findings suggest that tailored interventions, such as improving access to facilities and offering more flexible scheduling, are needed. Furthermore, by recognizing the specific motivational profiles and fields of study present, we can create more effective strategies to encourage PA, ultimately enhancing student health and well-being.

### 4.2. Strengths and Limitations

Among its strengths, this study addresses a critical topic: PA engagement among university students and its contribution to health. Furthermore, this research offers a comprehensive view by examining both motives and barriers, and by including an analysis of gender differences and a range of faculties. This enhances the relevance of the findings within the university context. The use of internationally validated instruments is another strength, facilitating a detailed understanding of the different motives for and barriers to PA participation. Importantly, our findings both confirm and contrast with previous research, adding nuance to the literature and highlighting the specific context of Libyan students.

However, it is important to note the limitations of this study. First, the classification of participants as ‘physically active’ or ‘inactive’ was based on a single, self-reported screening question. While this was a practical approach for survey allocation, it is recognized that participants’ understanding of what constitutes PA may vary, introducing potential misclassification bias. Future research would benefit from using standardized instruments like the International Physical Activity Questionnaire (IPAQ) for initial group classification. Additionally, while we used internationally validated questionnaires, they were not formally pre-tested within our specific Libyan student population prior to this study. Although the Arabic translation of the EMI-2 was available and used, future research could benefit from a pilot testing phase to ensure optimal cultural and contextual adaptation. Moreover, as this study was conducted at a single university in Tripoli and utilized a convenience sampling method, the findings may not be fully generalizable to all university students in Libya. Therefore, the generalizability of the results is limited, and future research should include a multi-center, random sampling approach to enhance representativeness.

Further, female participants were overrepresented in the sample, which may influence the results and interpretations, which limits the generalizability of our results. On the other hand, this may not be the case, as the barrier group had significantly more females than males, whereas the motive group had a greater number of males. This discrepancy could reflect differences in physical fitness or levels of participation in PA between genders. Moreover, this study did not take into account participants’ health status, which could influence the broader applicability of the findings and may neglect important barriers faced by individuals with chronic illnesses or disabilities. Additionally, as the data are self-reported measures, there is a possibility of social desirability bias and inaccuracies in their PA levels. Finally, since this study was conducted in Tripoli, Libya, it may not entirely reflect the experiences of university students in other cultural or geographical contexts

## 5. Conclusions

In conclusion, this study reveals the complex relationship between what motivates university students in Libya to engage in PA and the barriers they face. While many students are driven by the desire for better health and vitality, they often struggle with external challenges like limited time and resources. Gender differences also significantly influence this dynamic: male students are motivated by enjoyment and competition, whereas female students tend to prioritize weight management and face greater overall obstacles.

To address the low levels of PA among students, it is essential to create supportive environments that facilitate active lifestyles. This includes improving access to exercise facilities and offering more flexible program schedules. By understanding the unique needs of different student groups, universities can implement targeted strategies that encourage a more active lifestyle. Future research should build on these findings to develop and test tailored interventions.

## Figures and Tables

**Table 1:** Demographic characteristics of the Participants.

Variable	Description	Whole Sample		Motives Group		Barriers Group	
		*n* = 678	%	*n* = 303	%	*n* = 465	%
Age	<20 years	120	15.6%	43	14.2	77	16.6
	20–25 Years	599	77.9%	236	77.9	362	77.8
	>25 years	49	6.38%	24	7.9	26	5.6
Sex	Males	272	35.4%	171	56.4	101	21.7
	Females	496	64.6%	132	43.6	364	78.2
BMI	Underweight	51	6.6%	22	7.3	48	10.3
	Normal	530	69%	213	70.3	298	64.1
	Overweight	138	18%	53	17.5	85	18.2
	Obese	49	6.4%	15	4.9	34	7.3
Faculty	Health Sciences	429	55.9%	154	50.8	273	58.7
	Humanitarian sciences	166	21.6%	77	25.4	90	19.4
	Science and Engineering	173	22.5%	72	23.8	102	21.9

**Table 2:** The motives for physical activity participation of the participants.

Motives Subscales	Mean ± SD					
	Whole Sample (*n* = 303)	Sex		Field of Study		
		Males (*n* = 171)	Females (*n* = 132)	FMS (*n* = 154)	FSE (*n* = 72)	FHS (*n* = 77)
Stress Management	3.46 ± 1.03	3.60 ± 1.07	3.51 ± 1.09	3.61 ± 1.06	3.62 ± 1.02	3.39 ± 1.17
Revitalization	3.93 ± 1.01	3.93 ± 1.03	3.93 ± 0.97	3.91 ± 1.01	3.87 ± 0.94	4.02 ± 1.05
Enjoyment	3.13 ± 0.97	3.35 ± 0.97 **	2.84 ± 0.90	3.03 ± 0.97	3.19 ± 0.90	3.27 ± 1.03
Challenge	3.06 ± 1.03	3.34 ± 1.00 **	2.70 ± 0.93	2.90 ± 1.04	3.24 ± 0.97 *	3.21 ± 1.00
Social Recognition	2.24 ± 1.09	2.56 ± 1.11	1.81 ± 0.90	2.00 ± 1.06	2.38 ± 1.07	2.60 ± 1.05 **
Affiliation	2.48 ± 1.08	2.82 ± 1.05 **	2.02 ± 0.93	2.21 ± 1.04	2.67 ± 0.96	2.82 ± 1.14 **
Competition	2.88 ± 1.35	3.35 ± 1.31 **	2.26 ± 1.11	2.64 ± 1.33	3.09 ± 1.28	3.15 ± 1.35 **
Health Pressures	2.21 ± 1.19	2.24 ± 1.18	2.16 ± 1.19	2.16 ± 1.19	2.21 ± 1.17	2.30 ± 1.18
Ill Health Avoidance	3.60 ± 1.07	3.64 ± 1.07	3.54 ± 1.05	3.54 ± 1.06	3.74 ± 0.99	3.56 ± 1.13
Positive Health	3.95 ± 0.96	3.98 ± 0.98	3.90 ± 0.91	3.91 ± 0.93	3.92 ± 0.87	4.04 ± 1.08
Weight Management	3.56 ± 1.04	3.44 ± 1.02	3.71 ± 1.02 **	3.58 ± 1.05	3.44 ± 0.99	3.65 ± 1.03
Appearance	3.28 ± 1.13	3.20 ± 1.11	3.38 ± 1.14	3.21 ± 1.19	3.28 ± 1.00	3.42 ± 1.13
Strength and Endurance	3.82 ± 1.03	4.10 ± 1.08 **	3.45 ± 1.03	3.71 ± 0.85	3.93 ± 0.58	3.92 ± 0.85
Nimbleness	3.56 ± 0.99	3.50 ± 0.98	3.62 ± 0.98	3.56 ± 1.01	3.45 ± 0.89	3.64 ± 1.02

Abbreviations: standard deviation (SD); FMS Faculty of Medical Sciences; FSE Faculties of Sciences and Engineering; FHS Faculty of Humanitarian Sciences; * *p* < 0.05; ** *p* < 0.01.

**Table 3:** Scores for barriers to physical activity participation of the participants.

Barriers Scores	Mean ± SD					
		Sex		Field of Study		
	Whole Sample (*n* = 465)	Males (*n* = 101)	Females (*n* = 364)	FMS (*n* = 273)	FSE (*n* = 102)	FHS (*n* = 90)
Lack of energy score	2.65 ± 0.98	2.50 ± 1.04	2.70 ± 0.96	2.66 ± 0.99	2.51 ± 0.99	2.73 ± 0.94
Lack of motivation score	2.71 ± 0.97	2.73 ± 1.03	2.71 ± 0.97	2.72 ± 0.99	2.61 ± 0.94	2.77 ± 0.96
Lack of self-confidence score	2.26 ± 0.86	2.15 ± 0.92	2.28 ± 0.85	2.24 ± 0.84	2.32 ± 0.96	2.23 ± 0.85
**Internal barriers**	2.54 ± 0.62	2.46 ± 0.89	2.56 ± 0.67	2.69 ± 0.73	2.56 ± 0.76	2.75 ± 0.66
Lack of resources score	3.22 ± 0.97	2.93 ± 1.03	3.22 ± 0.97 *	3.16 ± 1.00	3.43 ± 1.00	3.23 ± 0.86
Lack of time score	3.45 ± 1.07	2.98 ± 1.00	2.96 ± 1.03	3.02 ± 1.03	2.90 ± 1.07	2.86 ± 0.95
Lack of support score	2.96 ± 1.01	3.33 ± 1.02	3.50 ± 1.08	3.51 ± 1.07	3.53 ± 1.13	3.28 ± 1.02
**External barriers**	3.21 ± 0.69 **	3.08 ± 1.01	3.22 ± 0.57	2.98 ± 0.61	3.04 ± 0.61	2.90 ± 0.50

Abbreviations: standard deviation (SD); FMS Faculty of Medical Sciences; FSE Faculties of Sciences and Engineering; FHS Faculty of Humanitarian Sciences; * *p* < 0.05; ** *p* < 0.01.

## Data Availability

The original contributions presented in this study are included in the article. Further inquiries can be directed to the corresponding author.
